# The Effects of Psychological Skills Training for Archery Players in Korea: Research Synthesis Using Meta-Analysis

**DOI:** 10.3390/ijerph18052272

**Published:** 2021-02-25

**Authors:** Eui Jae Kim, Hyun Wook Kang, Seong Man Park

**Affiliations:** 1 College of Sport Science, Dankook University, Cheonan-si 31116, Korea; 4770423@hanmail.net; 2 College of Liberal Arts, Dankook University, Cheonan-si 31116, Korea

**Keywords:** archery player, psychological skills training, comprehensive analysis, meta-analysis, effect size

## Abstract

The purpose of this meta-analysis study was to investigate the effectiveness of psychological skills training interventions for archery players in Korea. A total of 17 research papers examining the effects of psychological skills training interventions were selected for data analysis. To analyze the data, the Comprehensive Meta-Analysis (CMA) software program was used. The results of this study were as follows: The overall effect size of psychological skills training interventions for archery players was ES = 0.469 (Hedges’ g), which was a small effect size. The major factors that influenced the effects of psychological skills training interventions for archery players appeared to be the player level and training period. In particular, adult players and longer periods of training had bigger effect sizes. The results of this study demonstrate that psychological skills training for archery players is effective and show that the player level and training period are crucial factors in the psychological skills training design. Based on these findings, some implications for future research are discussed.

## 1. Introduction

At this very moment, sports players across the globe are constantly striving to improve their skills in order to achieve good results in their selected sports events. In order to reach their desired goals, components such as physical factors, motor dynamics, psychological factors, and environmental factors must all somehow be harmonized. In particular, it has been found that many athletes who are progressing towards elite or professional sports are likely to see the levels of their physical factors and motor skills be standardized upwards; thus, in many cases, psychological factors play a key role in deciding whether they win or lose [1]. Therefore, it can be difficult to demonstrate having the best skills without having the ability to be aware of and control one’s mental state in unpredictable game situations where the outcome is uncertain. In this sense, the sports field also began to recognize psychology as an area of training [2].

The Korean government also includes psychological training in sports science support projects, and since 2015, regional sports science centers have been selected by provinces to provide psychological skills training for local student athletes and unemployed team athletes [3]. Psychological skills training (PST) refers to a technique or strategy for practicing and training psychological skills that are helpful for playing games with a positive attitude, along with the aim of improving performance. Specific examples of PST include image training, relaxation, goal setting, surrounding awareness, and routine setup [4].

Archery is a representative type of closed exercise, and it is a sport in which psychological factors act largely enough that it can be called a mental sport. In particular, archery is known to be more psychologically affected than sports in other environments [5], so the ability to properly be aware of and control one’s mental state is important.

Meanwhile, academia has been interested in grasping the effectiveness of archery players’ psychological skills training. Looking at the related studies, most of the studies that applied psychological skills training reported the effectiveness of this training [5,6,7,8,9,10,11,12]. However, the inconsistency in results of these individual studies suggests the need for a more comprehensive evaluation of the effectiveness of such training. In this context, it is necessary to integrate individual studies to verify the practical significance, in the sense of the effect sizes of the psychological skills training for archery players, and to explain the inconsistent results of these individual studies.

Therefore, this study intends to conduct a meta-analysis to comprehensively evaluate the psychological skills training effect on archery players. The purpose of this study is to estimate the magnitude of the effect of psychological skills training on archery players in order to verify the practical significance and to identify the modulating variables that can explain the inconsistent results of previous individual studies. These attempts are expected to present the direction of follow-up research on psychological skills training and to further provide useful data for designing psychological skills training in the field of archery instruction.

In view of the above description, the specific research questions to be presented in this study are as follows.

First, what is the magnitude of the overall effect of archery players’ psychological skills training on their outcomes?

Second, what are the variables that control the effect of psychological skills training on archery players?

## 2. Research Method

### 2.1. Collection and Selection of Data

In this study, data were collected from thesis and academic journal articles published in Korea so far (~2019). Since the purpose of this study was to comprehensively explain the effectiveness of psychological skills training for domestic archery players, and the target of analysis was selected as a domestic study, the databases were limited to those which identified Korean articles and dissertations to perform the meta-analysis. The data collection used the Academic Research Information Service [13], Nuri Media [14], Korean Academic Information [15], and The Korea Research Foundation [16] online academic databases, and the data were collected primarily by searching for keywords such as: “Archery”, “Psychological Skills Training”, “Goal Setting Training”, “Psychology Training”, “Relaxation Training”, and “Routine Training”. Then, the titles and abstracts were reviewed to see if there was any relevance to this study. If the title and abstract alone were not sufficient to judge the relevance of the data, the entirety of each paper was reviewed for the final selection of the data for the analysis. The data selection process was carried out independently by two researchers, and in the case of mutual disagreement, the final analysis target data were selected through sufficient discussion between the researchers. As a result, a total of 3467 studies were primarily searched through the domestic academic databases, and only studies that satisfied the following criteria were selected for the final meta-analysis target: first, a study targeting archery players; second, an experimental study that verified the effectiveness of psychological skills training; third, a study that selected anxiety, psychological skills, and performance as dependent variables; and fourth, statistical values that enabled us to calculate the effect size (i.e., papers presenting mean and standard deviation, *t*-value, *p*-value, etc.).

**Figure 1:**
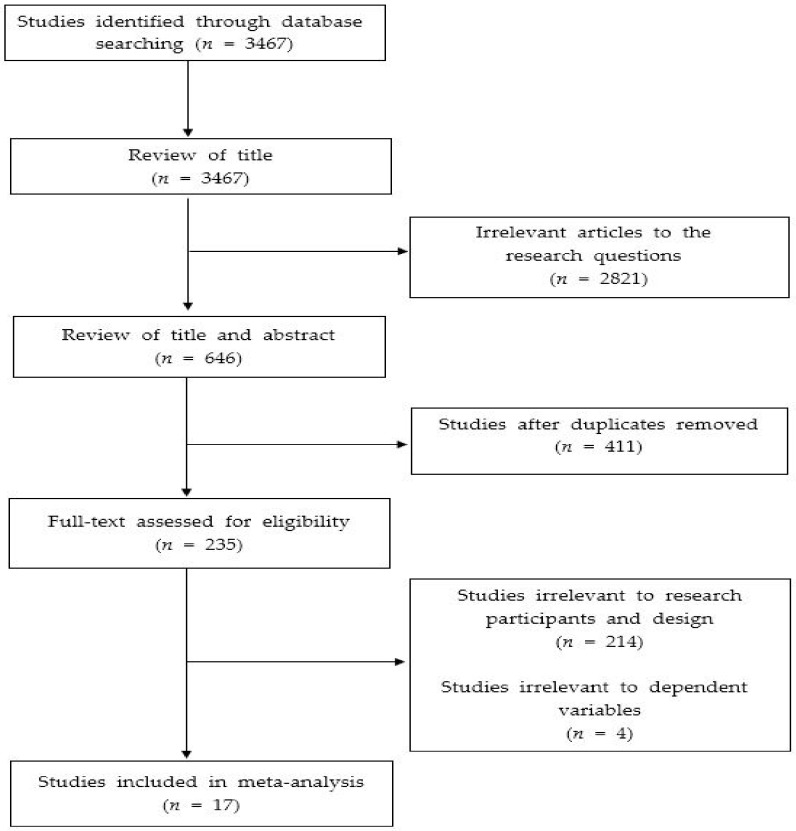
PRISMA flow chart.

It should also be noted that in the case of overlapping studies in which the thesis and the journal paper consisted of the same data, the journal paper was selected as an analysis target. As a result of reviewing the literature according to these data selection criteria, 17 studies were selected as the final meta-analysis target (Table 1), and the specific data collection and selection process is shown in Figure 1. Regarding the 14 academic articles chosen for analysis, a search of the Google Scholar electronic database [17] was conducted in order to secure sufficient quality of the data, with the search results confirming that all 14 articles could be accessed via the Google Scholar electronic database. With regard to the three dissertations used for this study, all of the dissertations were chosen from national universities in Korea. Overall, it is believed that the selected articles and dissertations are of sufficient quality.

### 2.2. Coding

The information necessary to calculate the effect size from the 17 studies selected as the final analysis target was entered in the coding form. Coding work was conducted by reflecting the opinions of one person who has performed psychological skills training research and one person who has performed meta-analysis research, and if there were differences between them, the coding items were modified and supplemented through continuous discussion. Meanwhile, the first (preliminary) coding was conducted by the lead author, and the recoding (secondary) was performed on the final analysis target, comparing and correcting any items that were inconsistent with the first coding data. Through this process, the final coding items were composed as shown in Table 2.

### 2.3. Data Analysis

#### 2.3.1. Analysis Tool

As a data analysis tool, the CMA (Comprehensive Meta-Analysis) Version 2.0 program (Biostat Inc., Englewood, NJ, USA) was used.

#### 2.3.2. Homogeneity Test

The homogeneity test was utilized to confirm whether the effect sizes extracted from the research can be considered to be extracted from the same population [18]. In this study, statistics were used as a method for verifying the homogeneity. The statistical value was only verified by the null hypothesis that the effect size of the population of the research was the same and was affected by the number of studies; thus, a statistical value representing the ratio of actual variance to total variance was additionally calculated [18]. In general, when the significance probability for the statistic is less than 0.10 and the statistic is more than 50%, the heterogeneity of the effect size was considered to be significant [19].

#### 2.3.3. Effect Size Calculation and Interpretation

To calculate the effect size, a standardized mean difference (Cohen’s d) calculation formula was used. Since Cohen’s d tends to overestimate the effect size when the sample size is small, the data were also converted into a corrected standardized mean difference (Hedges’ g) using the formula suggested by Hedges and Olkin [20]. According to the criteria of Cohen [21], the effect size was interpreted as a small size if it was between ES = 0.2 and ES = 0.5, a medium size if it was between ES = 0.5 and ES = 0.8, and a large size if it was ES = 0.8 or more, as shown in Table 3. In addition, another method of interpreting the effect size was used, where the effect size was analyzed by comparing the experimental group and the control group in the normal distribution curve by converting the effect size to the percentiles of non-overlap.

#### 2.3.4. Moderator Analysis

If the results of the homogeneity test and the heterogeneity statistics ($I^{2}$) showed that the size of the effects extracted from the research was heterogeneous, and the cause of the heterogeneity needed to be identified. In this study, a sub-group analysis was performed on the major variables suggested by the research to understand the cause of heterogeneity.

#### 2.3.5. Publication Bias

In order to secure the validity of the meta-analysis results, a publication bias test was conducted. Publication bias means that studies with positive findings account for a large proportion of studies subject to meta-analysis, resulting in an overestimation of the meta-analysis results [22]. In this study, the degree of bias was examined through a funnel plot and trim and fill.

#### 2.3.6. Independence Assumption and Analysis Unit Shift

When multiple results are reported in a single study, the assumption of independence can be problematic. Therefore, when estimating the total effect size, the problem of violating the assumption of independence was considered by using individual studies as an analysis unit [23].

## 3. Results

### 3.1. The Overall Effect Size on Psychological Skills Training of Archery Players

**Figure 2:**
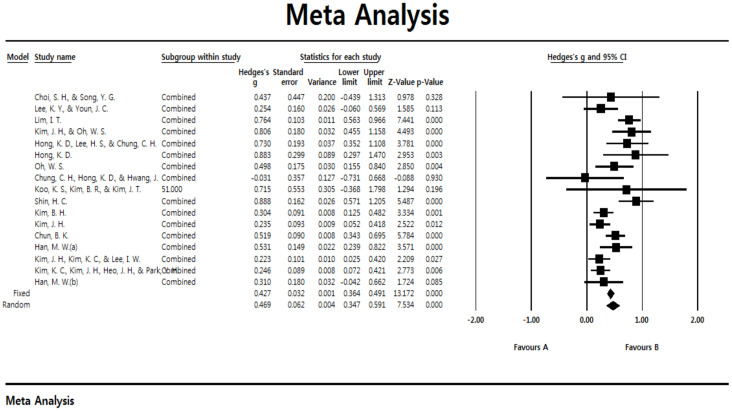
Forest plot of the summary statistics.

The results of analyzing the overall average effect size for psychological skills training of archery players are shown in Table 4. First, looking at the results of the homogeneity test, the value representing the ratio of the actual variance was as high as 66.442, and the Q value was 47.678, showing a notable difference at the significance level of 0.01 between the groups and the average effect size, and the effect sizes were found to be heterogeneous. Therefore, in this study, the total effect size was estimated using a random-effects model. As a result of the analysis, the overall average effect size was found to be ES = 0.469, and it was found to be significant in the 95% confidence interval. In addition, when the overall effect size was interpreted through the percentiles of non-overlap, assuming the control groups percentile was 50% in the normal distribution curve, the percentile of the experimental group was 68%, showing that the effects of the psychological skills training were more effective than for the non-experimental group by 18%. Meanwhile, a forest plot showing the summary statistics of the research subject is presented in Figure 2.

### 3.2. Analysis of Moderator Effect

In this study, a sub-group analysis was conducted on the main variables suggested in the research to identify the variables that most influence the effect of psychological skills training on archery athletes. The results of the moderation effect analysis are shown in Table 5.

As a result of analyzing the effect size by dividing the study subjects into adults and middle or high school students, it was found to be ES = 0.338 for adults and ES = 0.666 for middle or high school students. Each effect size was found to be significant in the 95% confidence interval, and the difference in effect size between regions was found to be statistically significant (Q = 11.326, df = 1, p = 0.001).

As a result of analyzing the effect size by dividing the training period into less than 12 weeks or over 12 weeks, it was found to be ES = 0.393 for under 12 weeks and ES = 0.581 for over 12 weeks. Each effect size was found to be significant in the 95% confidence interval, and the difference in effect size between regions was found to be statistically significant (Q = 5.027, df = 1, p = 0.025).

As a result of analyzing the effect size by dividing the training frequency into 1~2 times and 3 times or more, it was found to be ES = 0.428 for 1~2 times and ES = 0.637 for 3 times or more. Each effect size was found to be significant in the 95% confidence interval, and the difference in effect size between regions was not statistically significant (Q = 1.963, df = 1, p = 0.161).

As a result of analyzing the effect size by dividing the training time into less than 60 min or more than 60 min, it was found to be ES = 0.639 for less than 60 min and ES = 0.496 for more than 60 min. Each effect size was found to be significant in the 95% confidence interval, and the difference in effect size between regions was not statistically significant (Q = 1.157, df = 1, p = 0.282).

As a result of analyzing the effect size by dividing the publication type into academic journal articles and dissertations, it was found to be ES = 0.384 for academic journal articles and ES = 0.746 for dissertations. Each effect size was found to be significant in the 95% confidence interval, and the difference in effect size between regions was found to be statistically significant (Q = 12.948, df = 1, p = 0.000).

### 3.3. Publication Bias Verification

**Figure 3:**
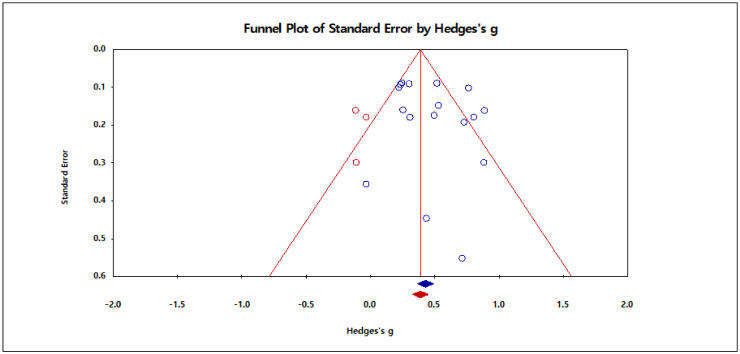
Funnel plot.

Meanwhile, a publication bias test was conducted to secure the validity of the meta-analysis results. First, as a result of visually examining the degree of bias through a funnel plot with the *x*-axis as the effect size (Hedges’ g) and the *y*-axis as the standard error, it was found that there was no excessive bias, as shown in Figure 3. As a result of examining the possibility of publication bias through trim and fill analysis in detail, as shown in Table 6, three studies were shown as correction values, and the effect size after the three studies were added was 0.398. Compared with the size of the effect before correction, it tended to decrease somewhat, but the difference was not found to be large.

## 4. Discussion

In this study, a meta-analysis was performed to comprehensively analyze the effectiveness of archery players’ psychological skills training.

First, as a result of comprehensively analyzing the effects of archery players’ psychological skills training, a statistically significant effect was confirmed. The results of this study imply that psychological skills training is an intervention that has a significant effect (i.e., ES = 0.469) on archery players. This will serve as an objective basis for more active use of psychological skills training as a training method to improve both the psychological changes and performance of archery players. Furthermore, this result strongly supports previous studies [24,25,26] that demonstrated the effectiveness of athletes’ psychological skills training through meta-analysis. On the other hand, the magnitude of the effect size of archery players’ psychological skills training on their outcomes was found to be only ES = 0.469. The results of this study can be compared with the recent meta-analysis study conducted by Kim Eui-jae and Kang Hyun-wook [25]. According to their research findings estimating the effect size of golf players’ psychological skills training on their outcomes, the authors [25] calculated the effect size was ES = 0.898, which was higher than the estimated effect size in this study. This suggests that even if the same psychological skills training is applied, the intervention effect may be different for archery players and golf players. In addition, these results strongly support the argument that the effects of psychological skills training may appear differently depending on the sport [25].

Second, the effect size of each athlete level was considered: it was higher in middle or high school athletes compared to adult athletes, and there was a statistically significant difference. This means that the heterogeneity between the sizes of the effects extracted from the analysis target study can be explained by the athlete level. It suggests that a greater effect can be expected when psychological skills training is applied to middle or high school athletes than to adult athletes. These results support a previous study [24] that observed that psychological skills training was more effective for amateurs or middle and high school golfers in comparison to pro players who have more experience. Moreover, it was found that adult athletes may have already experienced some psychological skills training and may have acquired psychological skills to some extent, so it was judged that there was no significant effect on the experiment. The fact that psychological skills training has a great effect on middle and high school athletes has important implications in the field of actual training for archery athletes. In particular, in the case of archery leaders who teach young athletes, if psychological skills training is introduced/implemented from an early stage, it will be of great help to improve athletes’ performance.

Third, when comparing the effect size by training period, it was found to be higher in the 12 weeks or more period than in less than 12 weeks period, showing a statistically significant difference. This means that the heterogeneity between the magnitudes of the effects extracted from the research can be explained by the training period, and it is possible to explain that a greater effect can be expected when psychological skills training is applied for a long time rather than a short period. According to Weinberg and Gould [27], in general, the effect of psychological skills training can be expected from at least 12 weeks, but it is difficult to say that the person is skilled in psychological skills just because this period has elapsed. In fact, they determined that it would take at least 6 months. Thus, it can be seen that psychological skills are not acquired through short-term experience but through long-term training and practice. Therefore, based on the results of this study, it is vital that archery leaders are aware of the importance of the length of the training period in the course of coaching their athletes, and also it is critical that they consider the training period when designing psychological skills training. However, considering that there is a variation in the number of cases of effect size between groups, caution is needed in the interpretation of the study results. In order to clearly grasp that the duration of training is a major variable controlling the effectiveness of psychological skills training, further studies to verify the effectiveness of psychological skills training should be accumulated.

Fourth, in the analysis of the effect size by training frequency, there was no significant difference between groups. This means that the heterogeneity between the effect sizes extracted from the analyzed study was not explained by the training frequency. These results support a previous study [16] that observed that training periods could better explain the effects of psychological skills training rather than the number of training sessions. On the other hand, it is important to note that when comparing the effect size between groups, it was relatively higher in the group that trained 3 or more times than the group that trained 1–2 times, even though there was no significant difference between groups in the analysis of the effect size by training frequency. Therefore, in the field of archery instruction, it would also be desirable to consider the frequency of psychological skills training along with the training period through a method of paralleling psychological skills training in the course of sports skills training.

Fifth, when comparing the effect size by training time, it was higher in less than 60 min training times compared to more than 60 min. The results of this study suggest that when psychological skills training is conducted for a long time, it may not have a significant effect. However, taking into account that there was not a significant difference in the magnitude of the effect between groups, and also that studies that conducted only one type of training and studies that performed multiple types of training were mixed in this analysis, caution in interpreting the study results is necessary. Although these limitations exist, this result is also worth considering in expecting high effectiveness in psychological skills training for archery players.

Sixth, when comparing the effect size of each publication type, it was higher in dissertations than in academic journal papers, and there was a significant difference between groups. This result implies that the heterogeneity between the sizes of effects extracted from the research analyzed can be explained by the type of publication, and it indirectly shows that there is no excessive bias in the studies included in this meta-analysis. Hwang [18] argued that if the research results are negative or contradictory to the expected results, it is highly likely that they are not published, and if the significance of the research results is high and significant research is likely to be published, there may be a problem of publication bias. In order to cope with this issue of publication bias, researchers will need to make an effort to share their research results with the academic community even if the effect on the experiment is not shown.

One regrettable aspect of the moderation effect analysis is that it was not possible to analyze the effect size for each type of psychological skills training individually. It was difficult to grasp the specific effects of each type of training because most of the studies analyzed were conducted with two or more psychological skills trainings at the same time, and the effects were verified accordingly. Although it should be considered that there are appropriate training types for each athlete, this study is considered necessary for a general discussion about which training types are more effective for archery athletes. One potential method to overcome this limitation is to divide the experimental group and apply different training types to observe the effects. For example, considering imagery training in group A, attention-intensive training in group B, routine training in group C, and evaluating the dependent variables can provide a basis for grasping the relative influence of each training type.

## 5. Conclusions

The purpose of this study was to examine the effectiveness of the psychological skills training for archery players and to identify the variables that influence the effect. To this end, a meta-analysis was conducted on 17 research papers that verified the effectiveness of psychological skills training for archery players. The following conclusions were drawn on the basis of the results of the analysis of the overall effect size and moderation effect of the psychological skills training according to the research problems set in this study. Firstly, psychological skills training is effective in improving the psychological changes and performance of archery players. Secondly, the player level and training period are the main variables that control the effect of psychological skills training.

Finally, the limitations of this study and suggestions for subsequent research are as follows. First, the purpose of this study was to comprehensively explain the effectiveness of psychological skills training for domestic archery players, and the target of analysis was selected as a domestic study. In follow-up studies, if a meta-analysis is performed on foreign research materials, a comparative analysis between domestic and foreign research will be possible, which is expected to be of academic value. In addition, it would be desirable to include a wider range of databases including Web of Science, the US National Library of Medicine, the Google Scholar electronic database, or other databases for further studies, so that a sufficient quality of the data can be better secured, and the results and implications can be applied to an international and wider range of contexts. Second, it is necessary to conduct a meta-analysis study on the effects of psychological skills training not only for golf and archery, but also for other sports. Psychological skills training has been used in various sports such as golf, archery, shooting, gymnastics, figure skating, taekwondo, and tennis. Therefore, if a meta-analysis study on psychological skills training for various sports is conducted, it will be helpful to present guidelines for psychological skills training programs by individual sports.

## Figures and Tables

**Table 1:** Lists of analyzed papers for meta-analysis.

Author	Publication Year	Publication Type	Sample Size
Choi & Song	2017	Article	8
Lee & Youn	2001	Article	30
Lim	2003	Dissertation	40
Kim & Oh	2017	Article	18
Hong, Lee & Chung	2007	Article	8
Hong	2008	Article	12
Oh	2015	Dissertation	20
Chung, Hong, & Hwang	2004	Article	8
Koo, Kim & Kim	2006	Article	12
Shin	1990	Dissertation	20
Kim	2007	Article	10
Kim	2005	Article	16
Chun	2005	Article	15
Han	1998	Article	16
Kim, Kim & Lee	2013	Article	8
Kim, Kim, Heo & Park	1999	Article	22
Han	1998	Article	10

**Table 2:** Example of the coding form.

Category
1. ID
2. Author
3. Title of paper
4. Publication year
5. Publication type
6. Sample size
7. Participants
8. Gender of Participants
9. Age of Participants
10. Type of PST
11. Training period
12. Training frequency
13. Hours per session
14. Dependent variable
15. Information of statistics

**Table 3:** Interpretation of effect size.

Effect Size (d)	Interpretation
0.2–0.5	Small size
0.5–0.8	Medium size
≥0.8	Large size

**Table 4:** Overall effect size.

Model	*n*	ES (U3)	95%CI	+95%CI	Heterogeneity			
					Q	df	*p*	$I^{2}$
Fixed	17	0.427 (66.5)	0.364	0.491	47.678	16	0.000	66.442
Random	17	0.469 (68.0)	0.347	0.591				

*n*: number studies, ES: effect size, CI: confidence interval.

**Table 5:** Sub-group analysis.

Sub-Group	n	ES	−95%CI	+95%CI	Q	df	p
Adults	67	0.338	0.230	0.446	11.326	1	0.001
Middle or High school students	36	0.666	0.508	0.823			
Less than 12 weeks	99	0.393	0.323	0.463	5.027	1	0.025
Over 12 weeks	26	0.581	0.432	0.730			
1 or 2 times	26	0.428	0.227	0.630	1.963	1	0.161
3 times or more	34	0.637	0.426	0.847			
Less than 60 min	28	0.639	0.421	0.856	1.157	1	0.282
More than 60 min	45	0.496	0.354	0.639			
Academic journal articles	100	0.384	0.300	0.469	12.948	1	0.000
Dissertations	25	0.746	0.568	0.923			

*n*: number studies, ES: effect size, CI: confidence interval.

**Table 6:** Analysis of trim and fill.

Value	Studies Trimmed	ES
Observed values	-	0.469
Adjusted values	3	0.398

## Data Availability

Publicly available datasets were analyzed in this study. This data can be found here: [http://www.riss.kr; http://www.dbpia.co.kr; http://kiss.kstudy.com; https://www.kci.go.kr; https://scholar.google.com].
